# A novel robot for imposing perturbations during overground walking: mechanism, control and normative stepping responses

**DOI:** 10.1186/s12984-016-0160-7

**Published:** 2016-06-11

**Authors:** Andrej Olenšek, Matjaž Zadravec, Zlatko Matjačić

**Affiliations:** University Rehabilitation Institute, Republic of Slovenia, Linhartova 51, Ljubljana, Slovenia

**Keywords:** Balance, Overground walking, Balance assessment robot, Pelvic manipulator, Rehabilitation robotics, Pelvis perturbations

## Abstract

**Background:**

The most common approach to studying dynamic balance during walking is by applying perturbations. Previous studies that investigated dynamic balance responses predominantly focused on applying perturbations in frontal plane while walking on treadmill. The goal of our work was to develop balance assessment robot (BAR) that can be used during overground walking and to assess normative balance responses to perturbations in transversal plane in a group of neurologically healthy individuals.

**Methods:**

BAR provides three passive degrees of freedom (DoF) and three actuated DoF in pelvis that are admittance-controlled in such a way that the natural movement of pelvis is not significantly affected. In this study BAR was used to assess normative balance responses in neurologically healthy individuals by applying linear perturbations in frontal and sagittal planes and angular perturbations in transversal plane of pelvis. One way repeated measure ANOVA was used to statistically evaluate the effect of selected perturbations on stepping responses.

**Results:**

Standard deviations of assessed responses were similar in unperturbed and perturbed walking. Perturbations in frontal direction evoked substantial pelvis displacement and caused statistically significant effect on step length, step width and step time. Likewise, perturbations in sagittal plane also caused statistically significant effect on step length, step width and step time but with less explicit impact on pelvis movement in frontal plane. On the other hand, except from substantial pelvis rotation angular perturbations did not have substantial effect on pelvis movement in frontal and sagittal planes while statistically significant effect was noted only in step length and step width after perturbation in clockwise direction.

**Conclusions:**

Results indicate that the proposed device can repeatedly reproduce similar experimental conditions. Results also suggest that “stepping strategy” is the dominant strategy for coping with perturbations in frontal plane, perturbations in sagittal plane are to greater extent handled by “ankle strategy” while angular perturbations in transversal plane do not pose substantial challenge for balance. Results also show that specific perturbation in general elicits responses that extend also to other planes of movement that are not directly associated with plane of perturbation as well as to spatio temporal parameters of gait.

## Background

Primary goal of walking is transferring the center of mass (CoM) between initial and target positions. While there exist many different forms of movement in nature only humans have been able to develop bipedal gait in their evolution. From biomechanical point of view bipedal gait is considerably demanding. Large portion of body mass is located high above the walking surface [1], and is supported by two legs that constitute relatively small base of support. Mastering proper coordination of such demanding locomotion apparatus that ensures good balance during walking also in presence of unexpected disturbances (e.g. slip, misstep, push...) requires good cooperation between biomechanical and neurophysiological processes.

However biomechanical and neurophysiological processes that underlay well balanced bipedal walking are complex and relatively unknown. The lack of deeper understanding of balance mechanisms may limit the potential of neurorehabilitation. Neurological injury of motor cortex (e.g. after stroke) often significantly hinders motor functions which inevitably causes balance problems. Nevertheless human brain can to some extent recover by bypassing damaged area and by re-establishing neural connections in neighbouring areas (neuroplasticity) [2–4] and in this way set up the conditions that provide the potential to restore gait function. Since the human brain is most susceptible to such recovery early after neurological injury, rehabilitation success depends on early start and patient specific therapeutic program. There is a strong initiative in the field of neurorehabilitation to develop novel training paradigms that would appropriately address also balance of bipedal gait.

The most common approach to studying balance is by applying unexpected perturbations to humans while they walk to cause kinematic and dynamic deviations that central nervous system addresses with appropriate motor responses [5–10]. Depending on the body segment that the perturbation acts upon we differentiate between distal and proximal perturbations. Distal perturbation applied base-of-support displacement at feet by suddenly moving the platform under stance leg [5, 6, 11] in medio/lateral or anterio/posterior direction to mimic slip [7, 12–15] in frontal or sagittal plane respectively. Alternatively subjects may be exposed to sudden obstacles in the line of walking to mimic tripping [8–10, 16] or they need to negotiate sinking platform to mimic misstep [17]. These experiments are almost exclusively conducted during overground walking. Primary concern of these paradigms is investigation of compensatory postural responses after inducing trips or slips as slips and trips are leading cause of falls and associated injuries in older adults. However if exposed to reoccurring slipping or tripping situations younger and older adults can improve compensatory responses associated with frequency as well as number and length of compensatory steps and time needed to stabilize after perturbation and reduces the incidence of falls [12–16]. In contrast, gait rehabilitation and balance recovery in neurologically impaired subjects do not target specific situations such as trip or slip (although trip and slip are great concerns in any impaired gait) but concern primarily with fundamental mechanisms associated with walking like forward propulsion, weight transfer, cyclical leg movement and dynamic balance that deteriorated due to changed muscle control after neurological damage. One particular concern associated with simulating slip is that moving platforms that are used to apply slipping perturbation [5, 6] actually deliver two successive perturbations, first one originating from platform acceleration and second one associated with platform deceleration which may be difficult to interpret as both elicit compound responses. For this reason single force proximal perturbations at pelvis are in gait rehabilitation after neurological damage more suitable as they evoke more elementary postural mechanisms as opposed to complex compound mechanisms. Only few studies have investigated dynamic balance responses originating from proximal perturbations in frontal plane in neurologically healthy population walking on a treadmill. Authors have shown that the majority of balance activities after lateral perturbation may be attributed to “stepping strategy” which depending on perturbation direction can manifest as “inward strategy” or “outward strategy” [18, 19]. Perturbation apparatus was rather simple pneumatically activated single degree of freedom (DoF) mechanism which may not be suitable for accessing dynamic balancing responses also in neurologically impaired population. There is a strong need for multi-DoF pelvis manipulation mechanism that would be able to follow natural pelvis movement during walking and that could also deliver well defined proximal perturbations in the transversal plane. Additionally, such pelvis robot would also need to be able to provide assistive force field to the pelvis to provide adequate support in walking of neurologically impaired population. Different approaches have been proposed to address these needs. Pneumatic actuation was used in [20] where three pneumatically driven degrees of freedom (DoF) were combined with two passive DoF providing five DoF in pelvis (three translations and two rotations). Linear electromagnetic actuation was implemented in the system [21] that had the capacity to deliver active force field in horizontal plane. System designed in [22] conceived lightweight and modular design composed of remote motors, pulleys, force sensors and cables that connect to selected attachment points on the hip brace to interact with pelvis movement. All three solutions were developed for use in combination with treadmill. Another important aspect when addressing dynamic balance during walking relates to walking conditions. So far studies that investigated balance mechanisms in relation to proximal perturbations during walking were conducted while walking on treadmill which may differ from balancing during overground walking.

The goal of our work was to develop a balance assessment robot (BAR) that could be used for studying balance responses during overground walking and would be able to deliver desired force field to the pelvis of a walking subject. In this paper we first present mechanics and control approach of BAR and explore their characteristics. Secondly, BAR was used to assess normative balance responses in a group of neurologically healthy individuals that may serve as a reference in subsequent studies in neurologically impaired population.

## Methods

### Mechanical design

BAR is composed of two primary subsystems: i) mobile platform (MP) and ii) pelvic manipulator (PM). Primary aim of mobile platform is to provide overground mobility in two DoF (forward movement and turning) and to ensure rigid support basis and appropriate attachment locations for the pelvic manipulator.

**Fig. 1:**
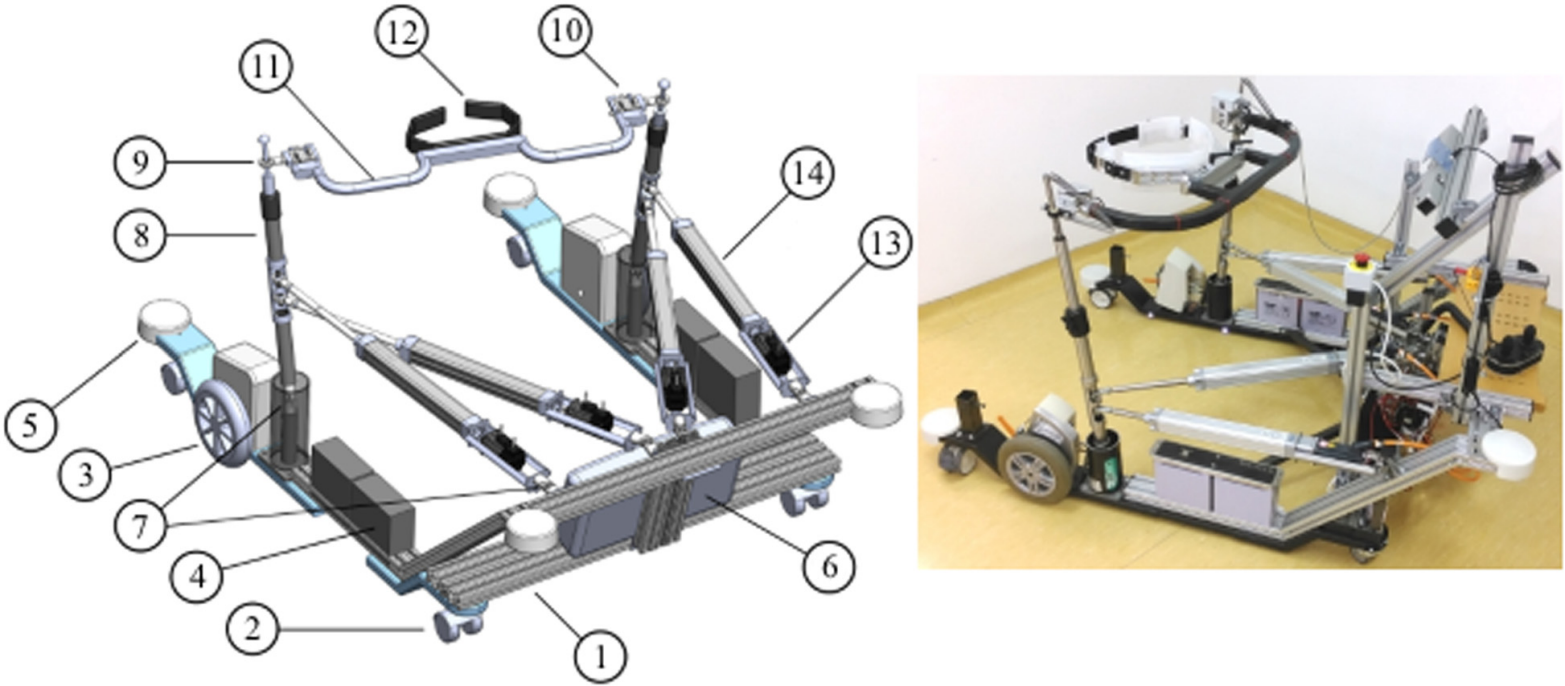
BAR - mechanical design and the actual system. *Left:* Detailed mechanical design of balance assessment robot. Mobile platform (MP): 1 - mobile platform frame, 2 - castor wheels, 3 - drive motors, 4 - batteries, 5 - bumper, 6 - control unit (Beckhoff PLC CX5020, Beckhoff Automation GmbH & Co. KG). Pelvis manipulator (PM): 7 - universal joint, 8 - vertical rod, 9 - spherical joint, 10 - pair of angularly displaced force sensors, 11 - pelvis element (PE), 12 - pelvis brace (PB), 13 - servo motor (Beckhoff AM122-F020, Beckhoff Automation GmbH & Co. KG), 14 - linear bearing (CASM-40-BS-0300AA-000, SKF Actuation Systems). *Right:* the actual system

MP is designed as U-shaped rigid steel frame with steel angular reinforcements designed to sustain loading associated with delivering perturbations. Within U-shaped rigid frame MP provides approximately 1.05 m of free space in medio/lateral direction and approximately 1.15 m of free space in anterior/posterior direction for unrestricted foot placement during walking. It is supported at the front with two castor wheels at left and right side respectively that enable angular motion of the mobile platform and two motorized wheels that are positioned at such location so that the line connecting their axes is aligned as close as possible with frontal plane aspect of the subject. In this arrangement the subject may turn at spot without having the need to step forward or backward. There are six universal joints located on the steel frame that further connect to PM. Two universal joints are located in the cylinders on the left and right side of the MP and connect to vertical rods of the PM. The remaining four universal joints are located at the front of the MP frame and connect to distal ends of linear actuators of PM. Linear actuators are composed of DC motors with absolute encoders that connect to linear unit (ball screw). The proximal ends of the linear actuators are connected to vertical rods of PM via spherical ball joints so that the left pair of linear actuators connect to vertical rod on the left and the right pair of linear actuators connect to vertical rod on the right. When actuated each pair of linear actuators deliver two DoF actuated movement to vertical rod it is connected to. At the top both vertical rods are connected by pelvic element (PE) with pelvic brace (PB) via spherical ball joints that are kept free to slide along the narrower end of both vertical rods. PE is designed as an arc with PB’s center positioned being approximately aligned with the center of arc’s curvature in the middle of both ends. In this way BAR provides sufficient empty space on both sides of PB and within PE so that the arms are free to swing and ensures alignment of CoM and the center of PE. Both ends of PE are equipped with a pair of perpendicularly arranged load cells that are on the inside attached to pelvic tubing made of carbon fibres i.e. PE and PB. When pelvis is tightly embraced each pair of load cells measures interaction forces between the subject’s pelvis and the PM in anterior/posterior (AP) and medio/lateral (ML) direction:

1 $$\begin{array}{@{}rcl@{}} F_{AP} = F_{1} + F_{4} \\ F_{ML} = F_{2} - F_{3} \end{array} $$

where *F*_1_ and *F*_4_ represent forces as measured by load cells at left and right ends of PE in AP direction and *F*_2_ and *F*_3_ represent forces as measured by load cells at left and right ends of PE in ML direction. Detailed composition of BAR and the actual system are presented in Fig. 1.

**Fig. 2:**
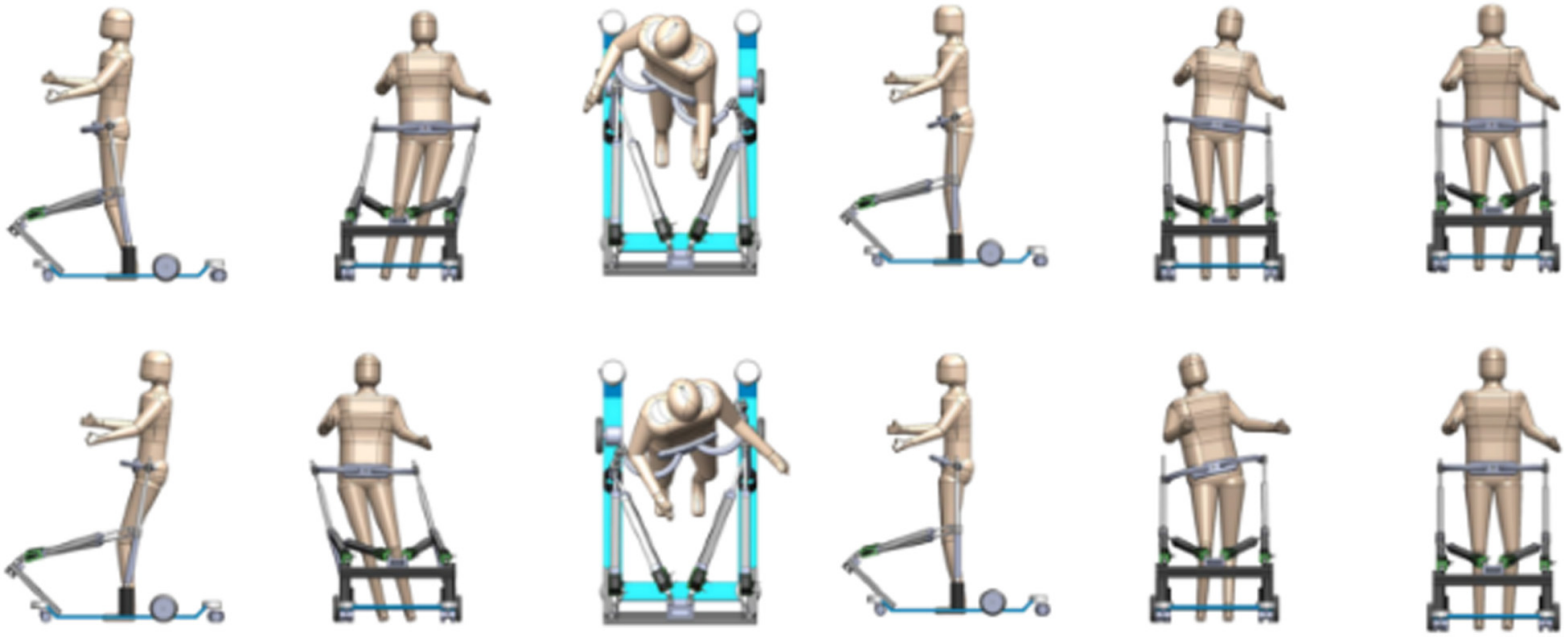
Pelvis DoFs. Schematic representation of available DoFs in pelvis when walking within BAR. From left to right: actuated forward(above)/backward(below) displacement, actuated left(above)/right(below) displacement, actuated CW(above)/CCW(below) rotation, passive anterior(above)/posterior(below) tilt, passive up (above)/down(below) obliquity, passive down(above)/up(below) displacement

Altogether PM alone provides six DoF movement: i) four DoF from vertical rods are diminished by one DOF due to PE connecting both tops to finally provide actuated pelvis AP displacement, actuated pelvis ML displacement and actuated pelvis rotation, ii) sliding motion of spherical ball joints extends three active DoF with three passive DoF i.e. pelvis tilt in sagittal plane, pelvis list in frontal plane and passive pelvis vertical displacement. All DoF are schematically presented in Fig. 2.

### Control

The overall control of the BAR is divided into two separate control strategies: i) control of spatial mobility for the MP and ii) haptic control of PM. Control of spatial mobility of MP is trivial; the operator selects desired linear and angular velocities of the MP via joystick and relays the information to control unit that calculates appropriate reference angular velocities of both drive motors. These are finally supplied to drive motor controllers (SDC2160N, Roboteq, Inc) that execute closed-loop velocity control. Haptic control of the PM on the other hand required development of suitable kinematic and kinetic frameworks of the conceived mechanism as well as the application of admittance-based haptic interaction control of forces between the subject’s pelvis and PE of the PM. In addition, control scheme needed to incorporate also possibility of imposing perturbation forces.

#### Kinematic framework

**Fig. 3:**
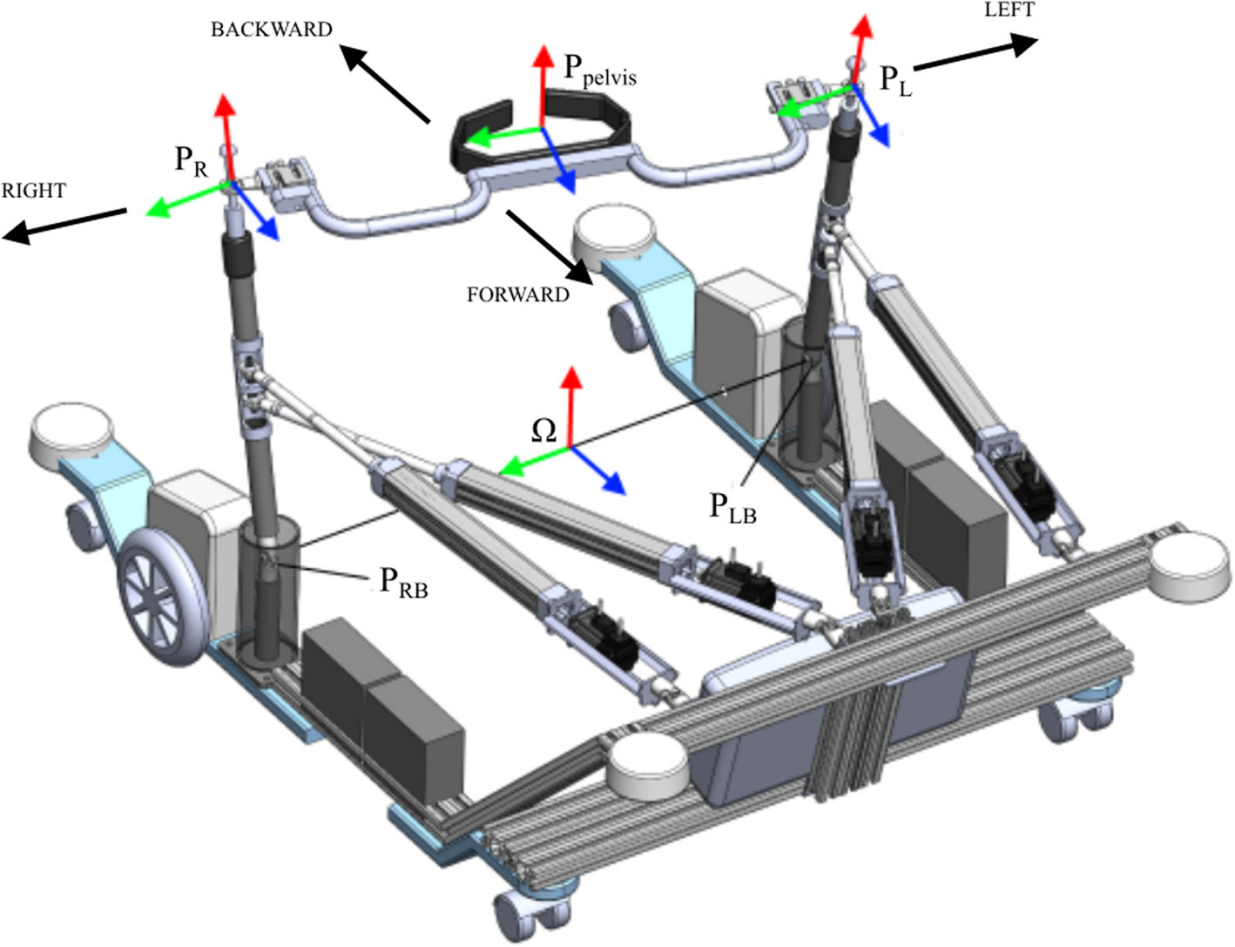
Kinematic framework of BAR. Schematic representation of selected locations on BAS that were used to develop kinematic model of BAR

Let *q*=[*q*_1_,*q*_2_,*q*_3_,*q*_4_] and $\dot {q}=\left [\dot {q_{1}}, \dot {q_{2}}, \dot {q_{3}}, \dot {q_{4}} \right ]$ be a set of positions and a set of velocities of linear actuators respectively. Let us further define such *Γ*_*L*_ and *Γ*_*R*_ so that the positions *P*_*L*_ and *P*_*R*_ with respect to coordinate frame of the BAR *Ω* (Fig. 3) can be expressed as

2 $$\begin{array}{@{}rcl@{}} P_{L} = \Gamma_{L}(q) \end{array} $$

3 $$\begin{array}{@{}rcl@{}} P_{R} = \Gamma_{R}(q). \end{array} $$

For left and right tops of vertical rods we define separate Jacobian matrices *J*_*L*_ and *J*_*R*_ so that

4 $$  \begin{array}{c} \dot{P_{L}}=\frac{dP_{L}}{dt}=\frac{d\Gamma_{L}}{dq}\cdot\dot{q}=J_{L}\cdot\dot{q}\\ \dot{P_{R}}=\frac{dP_{R}}{dt}=\frac{d\Gamma_{R}}{dq}\cdot\dot{q}=J_{R}\cdot\dot{q} \end{array}.  $$

Then the position of PE with respect to *Ω* is defined as

5 $$  P_{pelvis}=\left[ \begin{array}{c} x_{pelvis}\\ y_{pelvis}\\ z_{pelvis} \end{array} \right] = 0.5\cdot\left(P_{L}+P_{R}\right)  $$

and the corresponding rotation matrix (not taking into account the effect of passive DoF of PE) is given by

6 $$ R_{pelvis}(q) = \left[ \begin{array}{ccc} e_{x} & e_{y} & e_{z} \end{array} \right]  $$

where

7 $$ \begin{array}{c} e_{x} = \frac{P_{L}-P_{R}}{\left | P_{L}-P_{R}\right |}\\ e_{z} = \frac{0.5\cdot\left(P_{L}+P_{R}\right)-0.5\cdot\left(P_{LB}+P_{RB}\right)}{\left | 0.5\cdot\left(P_{L}+P_{R}\right)-0.5\cdot\left(P_{LB}+P_{RB}\right) \right |}\\ e_{y} = e_{z} \times e_{x} \end{array}  $$

and *P*_*LB*_ and *P*_*RB*_ denote the positions of left and right universal joints with respect to *Ω*. Pelvis rotation about z axis is then

8 $$ \Theta_{z}=arctan\left(\frac{R_{2,1}\cdot R_{3,3}-R_{2,3}\cdot R_{3,1}}{R_{3,3}\cdot R_{2,2}-R_{2,3}\cdot R_{3,2}} \right).  $$

By taking into account (4) and (5) pelvis velocity is then

9 $$ {}\dot{P}_{pelvis}\,=\,\frac{dP_{pelvis}}{dt}\,=\,0.5\cdot\!\left(\dot{P}_{L}\,+\,\dot{P}_{R}\right)=0.5\cdot\left(J_{L}\,+\,J_{R}\right)\cdot\dot{q}\,=\,\! \left[\! \begin{array}{c} J_{x}\\ J_{y}\\ J_{z} \end{array} \!\right]\cdot\dot{q}.  $$

Pelvis angular velocities are related to rotation matrix as

10 $$ \left[ \begin{array}{ccc} 0 & -\omega_{z} & \omega_{y}\\ \omega_{z} & 0 & -\omega_{x}\\ -\omega_{y} & \omega_{x} & 0 \end{array} \right] = \frac{dR(q)}{dt}R(q)^{T}  $$

where

11 $$ \frac{dR(q)}{dt}= \left[ \begin{array}{ccc} \dot{e}_{x} & \dot{e}_{y} & \dot{e}_{z} \end{array} \right]  $$

and

12 $$ \begin{array}{c} \dot{e}_{x} = \frac{de_{x}}{dq}\dot{q}\\ \dot{e}_{y} = \frac{de_{y}}{dq}\dot{q}\\ \dot{e}_{z} = \frac{de_{z}}{dq}\dot{q} \end{array}.  $$

Therefore, pelvis angular velocity about z axis is

13 $$\begin{array}{@{}rcl@{}} \omega_{z}=\dot{e}_{x,2}\cdot e_{x,1} + \dot{e}_{y,2}\cdot e_{y,1} + \dot{e}_{z,2}\cdot e_{z,1}&=&\frac{de_{x,2}}{dq}\cdot\dot{q}\cdot e_{x,1}+ \\+\frac{de_{y,2}}{dq}\cdot\dot{q}\cdot e_{y,1}+\frac{de_{z,2}}{dq}\cdot\dot{q}\cdot e_{z,1}&=&J_{\omega}\cdot\dot{q} \end{array} $$

Since PE has constant width the following kinematic constraint is valid

14 $$  \begin{array}{l} D = \left |P_{L}-P_{R} \right | = const \\ \dot{D}=\frac{dD}{dq}\cdot\dot{q} = J_{D}\cdot\dot{q} \end{array}.  $$

#### Kinetic framework

##### Force sensing

The interaction forces between the user and the PE of PM are being measured with two pairs of perpendicularly arranged load cells that are mounted on both ends of PM as shown in Fig. 1. When taking into account all measured forces *F*_1_, *F*_2_, *F*_3_ and *F*_4_ the interaction forces in X and Y direction as well as interaction moment around Z axis that the user imposes on PE are defined as

15 $$ \begin{array}{c} F_{x} = \left(F_{2}-F_{3}\right)\cdot cos\Theta_{z} - \left(F_{1}+F_{4}\right)\cdot sin\Theta_{z}\\ F_{y} = \left(F_{2}-F_{3}\right)\cdot sin\Theta_{z} + \left(F_{1}+F_{4}\right)\cdot cos\Theta_{z}\\ M_{z} = \frac{D}{2}\cdot \left(F_{1}-F_{4}\right) \end{array}.  $$

##### Control forces

Let *f*_*u*_=[*F*_*x*_,*F*_*y*_,*M*_*z*_]_*T*_ be a set of forces in *x* and *y* direction and moment around *z* axis respectively that the user applies on the PE. Let us further assume a constraint force *F*_*D*_=*F*_2_−*F*_3_ that the PE imposes to keep the distance between the tops of vertical rods fixed to the width of PE *D* (14). Introducing constraint force *F*_*D*_ ensures full rank of the Jacobian of PM given that four linear actuators were used to actuate only three DoF. Then the complete set of control forces is given by

16 $$ f_{c}=\left[ \begin{array}{c} f_{u}\\ F_{D} \end{array}\right] =\left[ \begin{array}{c} F_{x}\\ F_{y}\\ M_{z}\\ F_{D} \end{array} \right].  $$

##### External forces

In addition to control forces PM is able to deliver force within range from 0*N* to 300*N* in *x* and *y* directions and moment within range from 0*Nm* to 30*Nm* around *z* axis that the user would experience as an external force and moment, hence *f*_*ext*_. In the literature a considerable attention related to external forces imposed on the human during walking relate to perturbation forces and corresponding human reaction strategies that humans exercise to regain balance. Therefore, unless otherwise noted we will assume external forces to be perturbation forces and moment $f_{ext}= f_{p}={\left [{F_{x}^{p}},{F_{y}^{p}}, {M_{z}^{p}}, 0\right ]^{T}}$.

#### Admittance control

Let *x*=[*x*_*pelvis*_,*y*_*pelvis*_,*Θ*_*z*_,*D*]^*T*^ be a set of control states of the PE and $f_{ext} = f_{p}={\left [{F_{x}^{p}}, {F_{y}^{p}}, {M_{z}^{p}}, 0\right ]^{T}}$ a set of external perturbation forces. Therefore, total force acting on pelvis unit is a sum of individual components *f*=*f*_*c*_+*f*_*p*_. Admittance control law assumes second order relation between the state of PE *x* and forces *f* acting on it

17 $$ M\cdot \ddot{x}+B\cdot \dot{x}+K\cdot x= f_{c}+f_{p} = f  $$

where *M*, *B* and *K* represent the inertia, viscosity and the stiffness of the PM respectively. These parameters determine the quality of haptic interaction between the subject’s pelvis and the PM. Inertia matrix *M* determines inertia user experiences during movement whereas viscosity matrix *B* and stiffness matrix *K* define force and moment a subject would experience if an equivalent spring-damper module would be pushing the subject’s pelvis to neutral position. Ideally in transparent mode the space would feel empty and the PM would follow the subject’s pelvis movement without providing any resistance. This is only possible to some extent since by diminishing the inertia matrix *M* indefinitely the haptic interaction at some point becomes unstable. To compensate for the remaining inertia a compensatory force $M_{comp}\ddot {x}_{comp}$ was taken into account. Therefore the admittance equation is given by

18 $$ M\cdot \ddot{x}-M_{comp}\cdot \ddot{x}_{comp}+B\cdot \dot{x}+K\cdot x= f_{c}+f_{p} = f  $$

This directly yields

19 $$ \begin{array}{c} \ddot{x}=\frac{f}{M}+\frac{M_{comp}}{M}\cdot\ddot{x}_{comp}-\frac{B}{M}\cdot \dot{x}-\frac{K}{M}\cdot x\\ \dot{x}=\int\ddot{x}dt\\ x=\int\dot{x}dt \end{array}  $$

and is implemented as

20 $$ \begin{array}{c} \ddot{x}_{t}=\frac{f_{t}}{M}+\frac{M_{comp}}{M}\cdot\ddot{x}_{comp,t}-\frac{B}{M}\cdot \dot{x}_{t}-\frac{K}{M}\cdot x_{t}\\ \dot{x}_{t}=\dot{x}_{t-1}+\ddot{x}_{t-1}\cdot\Delta t\\ x_{t}=x_{t-1}+\dot{x}_{t-1}\cdot\Delta t\\ \ddot{x}_{comp,t}=\left(\dot{x}_{t}-\dot{x}_{t-1}\right)/\Delta t \end{array}  $$

where subscript *t* and *t*−1 denote current and previous time steps respectively. Parameters of admittance control were experimentally determined prior to our study and have not been changed thereafter. Their values are given in Table 1. Presented admittance control was developed in Beckhoff TwinCAT (Beckhoff Automation GmbH & Co. KG) software and was implemented in embedded PC CX5020 (Beckhoff Automation GmbH & Co. KG).

**Table 1:** Parameters of admittance control

DoF	M	M _comp_	$\text {B = 2}\sqrt {\mathrm {M}\cdot \mathrm {K}} $	K
X	0.5 kg	0 kg	7.1 kg/s	25 N/m
Y	0.8 kg	0 kg	8.9 kg/s	25 N/m
Z	0.12 kgm^2^	0.2 kgm^2^	0.49 Nm/rads	0.5 N/rad
D	1.0 kg	0 kg	3.16 N/m	2.5 N/m

### Experiment design

**Fig. 4:**
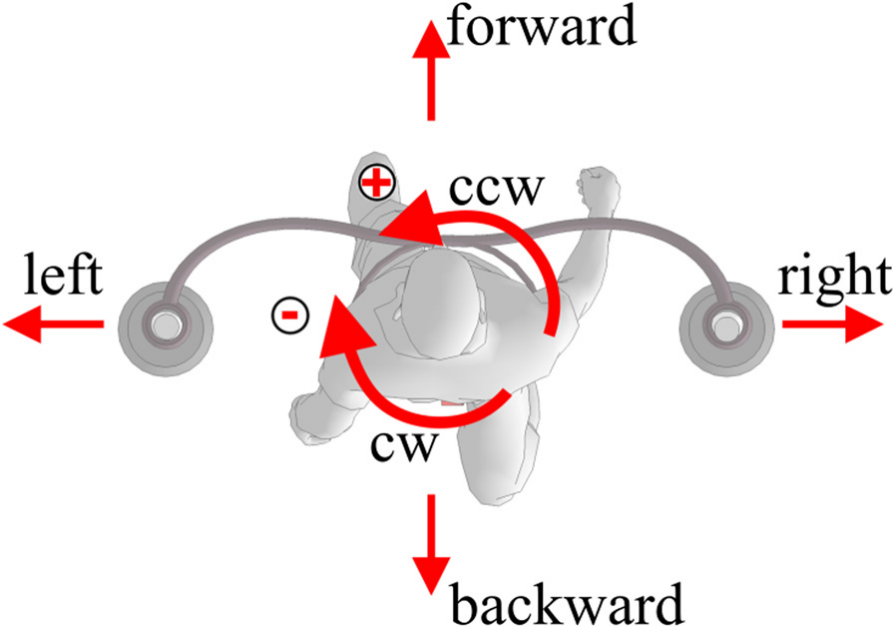
Perturbation directions. Schematic representation of perturbation directions with respect to human body

Candidates for this study were healthy adults with no known neurological or orthopaedical disorders and approximately average human body weight (80 kg) and body height (1.8 m) - anthropomorphic criteria were required to comply with the available range of pelvis brace height above walking surface (approximately between 80 cm and 95 cm) and with the available range of perturbation force/moment (approximately up to 300 N and 30 Nm). Seven subjects were selected (age: 33.4±8.5 years, body weight: 80.1±11.6 kg, height: 180.6±5.3 cm) to participate in a study where balancing responses to selected proximal perturbations were investigated. Given the design and control characteristics of the pelvic manipulator BAR is capable to deliver perturbations in all actuated DoF and in all of their combinations i.e. BAR is capable to deliver desired force and moment perturbations in transversal plane of subject’s pelvis. If we consider the available range of force/moment amplitudes the parameter space of available perturbations is enormous. For this reason we confined perturbation space to principal axes of human body (left/right - LR pelvis shift, forward/backward - FB pelvis shift and clockwise/counter clockwise - CW/CCW pelvis rotation). When selecting perturbations parameters our goal was to select such perturbation amplitude that would elicit substantial balancing responses while not creating fall threatening situations. Also shortest possible perturbation period was selected to avoid substantial responses before perturbation ended. Appropriate perturbation amplitude was experimentally determined and set to 15 % of bodyweight for LR and FB perturbations and to 1.5 % of bodyweight for CW/CCW rotation perturbations, where normalization ensured that all subjects were exposed to same similar accelerations during perturbation period. Similarly, perturbation period was experimentally determined and set to 150 ms which was the level at which perturbation of selected amplitude could be accurately and repeatedly delivered. Perturbation directions with respect to human body are shown in Fig. 4. Foot switch in left shoe was used for triggering perturbations in all selected directions at the time of left foot strike as well as for tracking the left foot contacts and stance phases.

Beside tracking pelvis position (and approximate position of CoM) through the kinematic model of PM and interaction forces between subject and PM, subject’s feet were equipped with reflective markers (medial malleoli, 1st metatarsal joint and 4th metatarsal joint) and BAR was equipped with Optitrack camera (NaturalPoint, Inc.) to also investigate foot placement strategies that associate with selected perturbations. Since the Optitrack camera was not aligned with the coordinate frame of BAR four additional markers were placed to a known positions on MP to determine transformation matrix between the coordinate frame of BAR and the Optitrack camera. We synchronized both systems by means of external trigger signal that aligned the start and the end of data recording in both subsystems.

At the beginning of session each subject completed approximately seven minute acclimation period when subject was given the opportunity to get acquainted with BAR and to experience perturbation forces/moments. For the first two minutes of acclimation period subject was walking without being perturbed. The reminder of acclimation period subject was subjected to perturbations in identical way as in subsequent experiment - perturbations of the same amplitude and duration as in subsequent experiment that were delivered randomly in selected directions and always at the time of left foot contact as well as in no less than six second intervals. At the end of acclimation period subject experienced each selected perturbation at least ten times. After acclimation period the subject was equipped with reflective markers and firmly fastened within PB. All subjects wore special belt that accommodates shape according to anthropomorphic characteristics of each subject’s pelvis and in this way ensures that subjects were evenly fastened around waist within PB while also inhibiting relative movement between subject’s pelvis and PB. This guaranties optimal force transmission between the subject and the PM which is imperative for proper operation of admittance control.

The experiment began with approximately one minute unperturbed walking period to obtain baseline data. Afterwards each perturbation was repeated five times where the sequence of perturbations was randomly generated prior to first perturbation. There was at least six seconds recovery period between two perturbations that allowed each subject to fully recover from perturbation, if necessary in several steps. Throughout the experiment each subject was given visual feedback on laptop screen that in real time graphically illustrated current pelvis position with respect to the center of available range of PM movement - due to limited stroke of linear motors PB displacement was limited to approximately 25 cm from center in each direction in transversal plane. During the first three seconds after perturbation onset the visual feedback was withdrawn by temporarily suspending graphical display of current pelvis position in order to leave the subject to cope with the perturbation and to respond solely to regain balance and not to worry about current pelvis position during response. Afterwards the subject was again presented with graphical display of current pelvis with respect to the center of available range of PM movement which the subject was instructed to consider as guidance to re-align pelvis position with the center of available range of PM movement. The recovery period of six seconds and its division into two halves was selected after we experimentally investigated the time needed to adequately respond to perturbations and to re-align pelvis position with the center of available range of PM movement. Walking velocity was set to 0.85 m/s primarily because this velocity represents a value at which stroke survivors are considered as community walkers [23]. This will enable use to refer to the results of this particular study in future experiments with stroke subjects. All subjects signed informed consent before testing and the experimental protocol was approved by the University Rehabilitation Institute institutional ethical committee.

### Data processing

In this study typical reference data assessed during walking in BAR without applied perturbations as well as a response to perturbation was in this study composed of pelvis movement in transversal plane (pelvis displacement in ML and AP directions and pelvis rotation about vertical axis), interaction forces/moment between subject and PM in transversal plane and stepping responses in terms of step length, step width and step time that were extracted from marker positions. First all data were segmented into strides where gait cycle was defined with two consecutive foot strikes of the same leg. Left foot strikes were determined by foot switch whereas the right foot strikes were determined as the local maxima of the ankle marker positions in the anterior/posterior direction [24]. Then linear length normalization was applied to convert the stride time axis to an axis representing percentage of stride so that in the duration of one stride 0 % represented the opening foot strike and 100 % represented the closing foot strike of stride. Since responses to some perturbations span over several steps all data were segmented into observation interval that covered second half of the stride that preceded the perturbation (at −50 %) followed by two and a half strides that started with perturbation at the time of left foot strike (at 0 %) and finished approximately at right foot strike at 250 %. For consistency, the same observation interval was considered also when determining reference natural gait without perturbations.

#### Responses in pelvis movement and interaction forces/moment

Pelvis movement in transversal plane (pelvis displacement in ML and AP directions and pelvis rotation about vertical axis in CW/CCW direction) and associated interaction forces/moment were obtained from movement of central point of PE (5) and forces as measured from two pairs of force sensors via (15) respectively. We assumed that if subject’s pelvis is properly fastened within the PB central point of PE and CoM are approximately aligned and there is no relative movement between that would cause any discrepancies in transferred forces between the subject and the PM. Representative reference values and responses to each perturbation for each subject (that span over selected observation interval) was then calculated by averaging across five trials.

#### Stepping responses

Stepping responses were investigated in terms of step lengths, step widths and step times. We calculated left (right) step length as AP distance between ankle markers at the moment of left (right) foot strike while left (right) step width was defined as the ML distance between the same markers at the moment of left (right) foot strike [24]. Similarly, left (right) step time was defined as the time between consecutive right(left) foot strike and left (right) foot strike. Since we assumed that balance reactions would span over selected observation interval single stepping response consisted of a series of alternating left and right step lengths, step widths and step times. For each subject series of steps related to the same perturbation (or unperturbed walking) were then averaged across five repetitions to obtain subject’s representative series of step lengths, step widths and step times for unperturbed gait and for each type of perturbed gait. Finally stepping responses were averaged across all subjects to obtain group series of step lengths, step widths and step times for unperturbed gait and for each type of perturbed gait. To determine whether selected perturbations had significantly affected stepping responses one way repeated measures ANOVA was conducted to separately compare step lengths, step widths and step time between successive steps in selected observation interval (one step prior and five steps following perturbation) separately for each experimental condition (normal walking and selected perturbations). Bonferroni adjusted post-hoc pairwise comparisons were conducted when a main effect or interaction was detected. The level of statistical significance was set to *P*<0.05. We used Shapiro-Wilk test of normality to verify normal distribution of data - in all experimental conditions *p* - value remained above the level of *p*>0.05 indicating normal distribution. To visually evaluate stepping responses footprints at left and right foot strikes were generated directly from averaged step lengths and step widths and time-aligned at the onset of perturbation i.e. at 0 %.

#### Data interpretation

All responses were interpreted from two perspectives: i) design and control characteristics of BAR and ii) response variations in relation to perturbation type. First, the degree of variability in responses was an indication whether BAR and accompanying control scheme could provide desirable control characteristics. Ideally when when walking within BAR the subject should feel no interaction forces between the pelvis and the PM that could condition subject’s behaviour during walking (except during perturbation period). If this was achieved subject’s responses would depend solely on subject’s internal mechanisms that subject typically applies when facing various experimental conditions. The degree of variability was assessed in terms of standard deviations of responses and qualitatively compared between different responses. Second, characteristics of responses were reviewed in relation to associated perturbation direction and the effects of selected perturbations on associated stepping responses were statistically evaluated.

## Results

### Perturbations in frontal plane

**Fig. 5:**
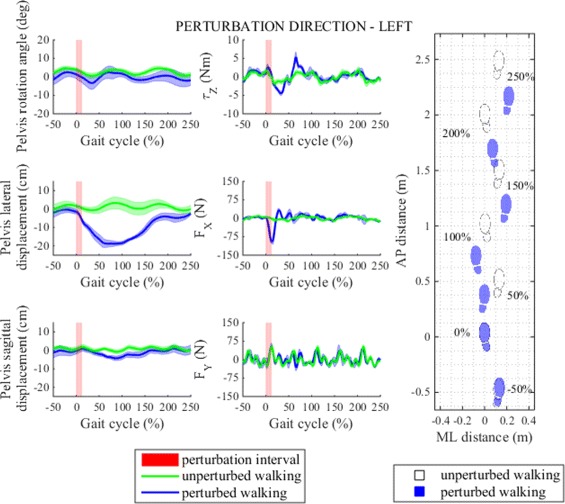
Perturbation to left direction. *Left* - pelvis movement and interaction forces/moment associated with actuated DoF in transversal plane for unperturbed and perturbed walking over selected observation interval for single typical subject was averaged across five single responses. *Right* - graphical illustration of foot placement at left (approximately at 0, 100 and 200 %) and right (approximately at −50, 50, 150 and 250 %) foot strikes for unperturbed and perturbed walking over selected observation interval for a group of subjects was generated from averaged group step lengths and step widths and time-aligned at the onset of perturbation at 0 %

Figures 5 and 7 show pelvis movement and interaction forces/moment as well as foot placement after perturbation was delivered in ML direction i.e. left/right. Both perturbations caused desired interaction forces during the perturbation period that enforced CoM displacement in the direction of the perturbation force. When perturbed to the left pelvis was displaced gradually over two steps (from 0 to 100 %) by approximately 20 cm to the left and gradually returned to neutral position by the end of the observation period. After perturbation to the right pelvis was displaced by approximately 10 cm to the right majority of which was achieved during the first stance phase of the right leg after perturbation (approximately from 50 to 100 %). Compared to perturbation to the left subjects recovered to normal pattern already in the next step. In sagittal plane a minor backward pelvis shift was recorded in both cases that however did not change alternating pattern of pelvis AP movement. On the other hand except from mild setback in CCW pelvis rotation after perturbation to the right none of the two perturbations evoked any substantial response in pelvis rotation.

**Fig. 6:**
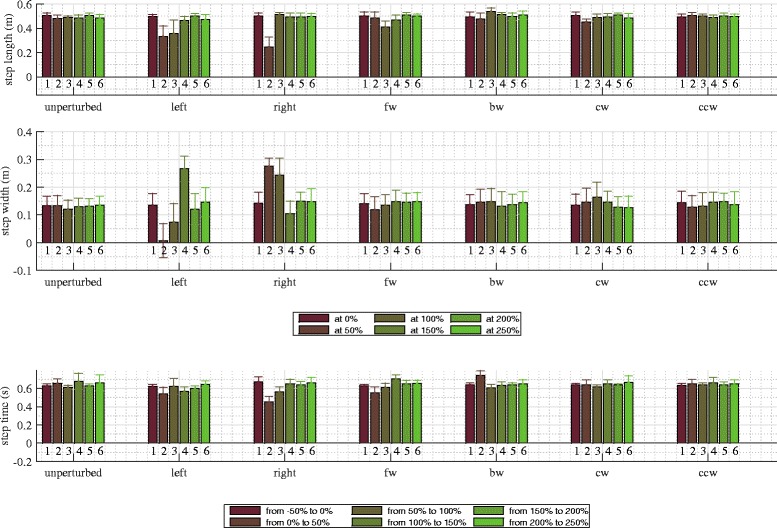
Stepping responses. Step length, step width and step time responses in unperturbed walking and after selected perturbations in transversal plane. Step length and step width responses correspond to distances between anterior and posterior ankle marker at the time of left (approximately at 0, 100 and 200 %) and right (approximately at 50, 150 and 250 %) foot strikes. Step time responses correspond to time intervals between consecutive foot off and foot strike of the same leg, i.e. left step times (approximately from −50 to 0 %, from 50 to 100 % and from 150 to 200 %) or right step times (approximately from 0 to 50 %, from 100 to 150 % and from 200 to 250 %) feet respectively

Perturbation in the left direction had substantial effect on stepping responses (Fig. 6). The first two steps after perturbation were considerably shorter than the remaining three steps. In their first step after perturbation subjects placed their right foot in front of their left foot, hence resulting in almost zero step width, in the next (left step) they increased step width by placing their left foot even more to the left but kept it still well below step width of unperturbed walking, finally they responded by placing the right foot far to the right thus increasing the step width to return to original line of walking and to restore normal step width thereafter. We also notice that the immediate (right) step after perturbation to the left was the fastest and that normal step time was restored early after perturbation. The effect of left perturbation on step length and step width is graphically illustrated with footprints in Fig. 5. Statistical analysis showed that after perturbation to the left step length, step width and step time responses in observation period changed significantly and post-hoc pairwise analysis found that statistically significant interactions between steps in observation period exist in step length and step width responses but not also in step time responses. Main effects and pairwise interactions are listed in Table 2.

**Fig. 7:**
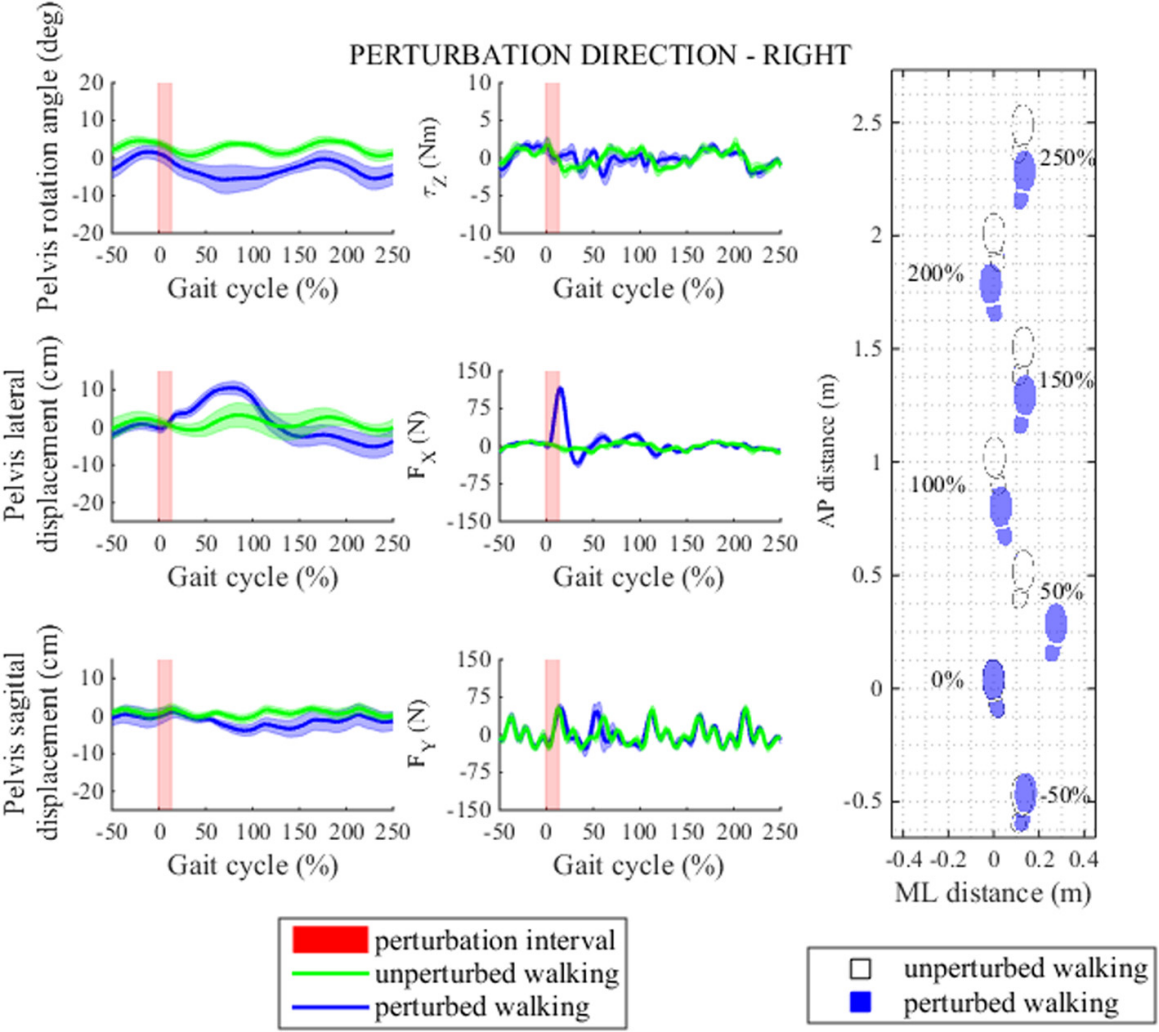
Perturbation to right direction. *Left* - pelvis movement and interaction forces/moment associated with actuated DoF in transversal plane for unperturbed and perturbed walking over selected observation interval for single typical subject was averaged across five single responses. *Right* - graphical illustration of foot placement at left (approximately at 0, 100 and 200 %) and right (approximately at −50, 50, 150 and 250 %) foot strikes for unperturbed and perturbed walking over selected observation interval for a group of subjects was generated from averaged group step lengths and step widths and time-aligned at the onset of perturbation at 0 %

**Table 2:** *F* - test values and *p* values from one way repeated measures ANOVA and Bonferroni adjusted post-hoc pairwise analysis on step length, step width and step time for unperturbed walking and for selected perturbations

			No perturbations	Left	Right	Forward	Backward	CW	CCW
Step length	Within - subjects effect	F - test	2.518	10.597	47.874	6.667	2.724	4.331	0.551
		*p* - value	0.051	0.000*	0.000*	0.000*	0.038*	0.004*	0.736
					1-2 (0.003*)				
				1-2 (0.034*)	2-3 (0.002*)	1-3 (0.055)		2-4 (0.004*)	
	Pairwise comparison - Step-Step (*p*-value)		/	2-5 (0.068)	2-4 (0.003*)	3-5 (0.074)	/	2-5 (0.013*)	/
				2-6 (0.033*)	2-5 (0.003*)	3-6 (0.085)			
					2-6 (0.004*)				
Step width	Within - subjects effect	F - test	0.826	38.288	43.304	2.793	2.426	8.021	1.328
		*p* - value	0.541	0.000*	0.000*	0.035*	0.058	0.000*	0.279
				1-2 (0.017*)	1-2 (0.001*)				
				1-4 (0.000*)	1-3 (0.046*)			1-3 (0.116)	
				2-4 (0.000*)	2-4 (0.000*)			2-3 (0.045*)	
	Pairwise comparison - Step-Step (*p*-value)		/	2-5 (0.020*)	2-5 (0.001*)	2-3 (0.195)	/	3-5 (0.040*)	/
				2-6 (0.018*)	2-6 (0.002*)			3-6 (0.136)	
				4-5 (0.002*)	3-4 (0.001*)				
				4-6 (0.003*)	3-6 (0.016*)				
					4-6 (0.009*)				
Step time	Within - subjects effect	F - test	1.778	5.046	21.077	11.346	12.662	1.373	0.734
		*p* - value	0.148	0.002*	0.000*	0.000*	0.000*	0.262	0.604
					1-2 (0.008*)	1-4 (0.063)	1-2 (0.068)		
					1-3 (0.099)	2-3 (0.058)	2-3 (0.026*)		
	Pairwise comparison -Step-Step (*p*-value)		/	2-6 (0.055)	2-3 (0.000*)	2-4 (0.041*)	2-4 (0.024*)	/	/
				4-6 (0.088)	2-4 (0.003*)	2-5 (0.062)	2-5 (0.063)		
					2-5 (0.011*)	2-6 (0.062)	2-6 (0.009*)		
					2-6 (0.001*)				

*statistically significant difference p < 0.05

In their response to perturbation to the right subjects considerably shortened the first step after perturbation (right step), they doubled their step width and almost halved step time by rapidly placing their right leg more outward (Fig. 6). In the following step they restored usual step length, they continued with somewhat shorter step time and increased step width by placing their left leg more outward and settled at approximately the same pace and line of walking as before perturbation. The effect of right perturbation on step length and step width is graphically illustrated with footprints in Fig. 7. Statistical analysis showed that after perturbation to the right step length, step width and step time responses in observation period changed significantly and post-hoc pairwise analysis found that statistically significant interactions exist between steps in observation period in step length, step width and step time responses. Main effects and pairwise interactions are listed in Table 2.

### Perturbations in sagittal plane

**Fig. 8:**
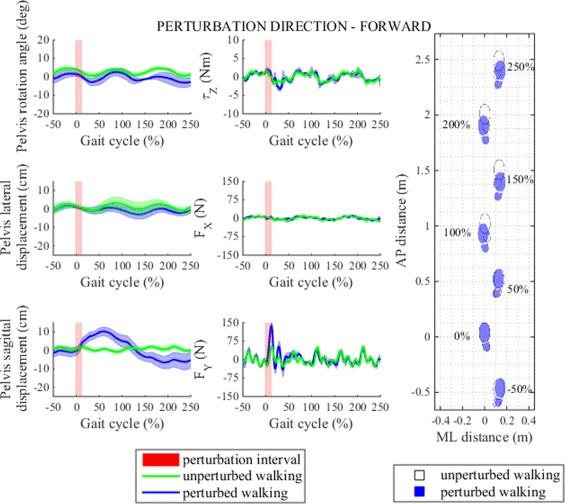
Perturbation to forward direction. *Left* - pelvis movement and interaction forces/moment associated with actuated DoF in transversal plane for unperturbed and perturbed walking over selected observation interval for single typical subject was averaged across five single responses. *Right* - graphical illustration of foot placement at left (approximately at 0, 100 and 200 %) and right (approximately at −50, 50, 150 and 250 %) foot strikes for unperturbed and perturbed walking over selected observation interval for a group of subjects was generated from averaged group step lengths and step widths and time-aligned at the onset of perturbation at 0 %

**Fig. 9:**
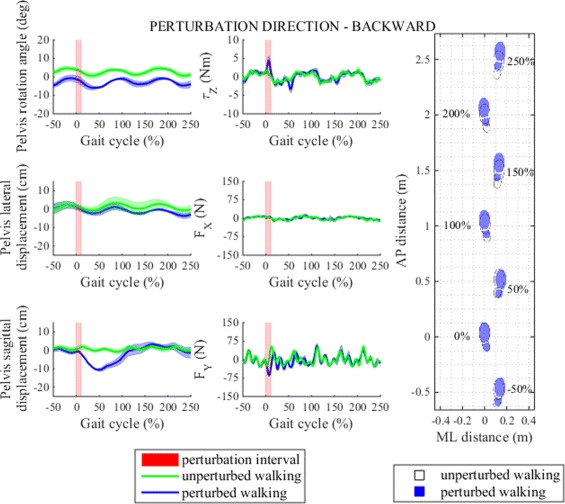
Perturbation to backward direction. *Left* - pelvis movement and interaction forces/moment associated with actuated DoF in transversal plane for unperturbed and perturbed walking over selected observation interval for single typical subject was averaged across five single responses. *Right* - graphical illustration of foot placement at left (approximately at 0, 100 and 200 %) and right (approximately at −50, 50, 150 and 250 %) foot strikes for unperturbed and perturbed walking over selected observation interval for a group of subjects was generated from averaged group step lengths and step widths and time-aligned at the onset of perturbation at 0 %

Figures 8 and 9 show pelvis movement and interaction forces/moment as well as foot placement after perturbation was delivered in AP direction i.e. forward/backward. We notice that both perturbations caused desired interaction forces in the AP direction during the perturbation period that enforced CoM displacement in the direction of perturbation force. When compared to unperturbed walking pelvis displacement increased by approximately 10 cm in forward direction when perturbed in forward direction and 10 cm in backward direction when perturbed in backward direction and took more than two gait cycles to recover. However neither forward or backward perturbation had any substantial effect on pelvis movement or interaction force in ML direction or pelvis rotation or interaction moment in transversal plane.

Both perturbations demanded adjustments in stepping responses (Fig. 6). After perturbation was imposed in forward direction the first two steps were slightly shortened; normal step length was recovered in the next two steps. Additionally, step width was slightly reduced in the first step immediately after perturbation which indicates somewhat more inward placement of the right foot; normal step width was recovered in the next two steps. Similar pattern was present in step time where we again recorded shorter step time in the step immediately after perturbation, in the following two steps step time was increased and exceeded step time of unperturbed walking and settled in the last two steps. The effect of forward perturbation on step length and step width is graphically illustrated with footprints in Fig. 8. Statistical analysis showed that after perturbation in forward direction step length, step width and step time responses in observation period changed significantly and post-hoc pairwise analysis found that statistically significant interactions between steps in observation period exist in step time response but not also in step length or step width responses. Main effects and pairwise interactions are listed in Table 2.

In response to backward perturbation subjects slightly decreased step length in the first step following perturbation then increased the next step length and gradually stabilized the following steps at approximately the same step length as in unperturbed walking. On the other hand step width shows very small increase in the first two steps after perturbation compared to unperturbed walking and only minor changes thereafter. Finally, step time rises to its maximal value in the first step after perturbation, decreases in the next step and then recovers to approximately the same step time as in unperturbed walking. The effect of backward perturbation on step length and step width is graphically illustrated with footprints in Fig. 9. Statistical analysis showed that after perturbation in backward direction only step length and step time responses changed significantly in observation period but not also step width responses and post-hoc pairwise analysis found that statistically significant interactions between steps in observation period exist only in step time responses but not also in step length responses. Main effects and pairwise interactions are listed in Table 2.

### Perturbations in transversal plane

**Fig. 10:**
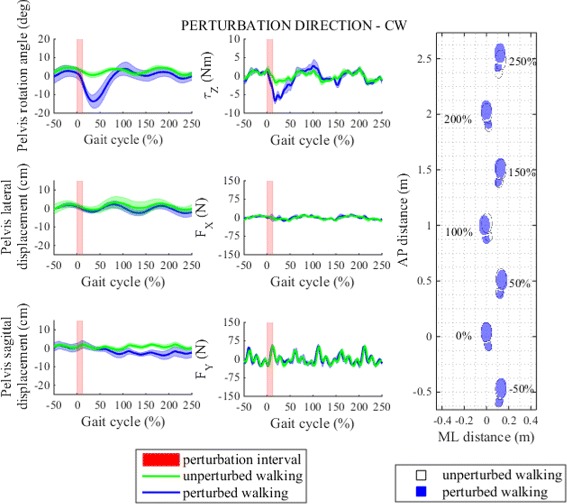
Perturbation to clockwise direction. *Left* - pelvis movement and interaction forces/moment associated with actuated DoF in transversal plane for unperturbed and perturbed walking over selected observation interval for single typical subject was averaged across five single responses. *Right* - graphical illustration of foot placement at left (approximately at 0, 100 and 200 %) and right (approximately at −50, 50, 150 and 250 %) foot strikes for unperturbed and perturbed walking over selected observation interval for a group of subjects was generated from averaged group step lengths and step widths and time-aligned at the onset of perturbation at 0 %

**Fig. 11:**
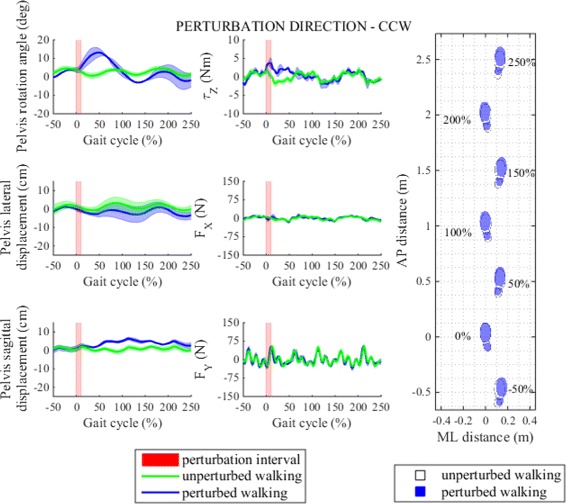
Perturbation to counter clockwise direction. *Left* - pelvis movement and interaction forces/moment associated with actuated DoF in transversal plane for unperturbed and perturbed walking over selected observation interval for single typical subject was averaged across five single responses. *Right* - graphical illustration of foot placement at left (approximately at 0, 100 and 200 %) and right (approximately at −50, 50, 150 and 250 %) foot strikes for unperturbed and perturbed walking over selected observation interval for a group of subjects was generated from averaged group step lengths and step widths and time-aligned at the onset of perturbation at 0 %

Figures 10 and 11 show pelvis movement and interaction forces/moment as well as foot placement after perturbation was delivered in CW/CCW directions. Both perturbations caused desired interaction moments during the perturbation period that enforced pelvis rotation in the direction of the perturbation moment. From pelvis rotation trajectory we see that the CW perturbation moment disturbed natural rotation movement of the pelvis - instead of continuing with alternating CW/CCW movement pelvis followed large perturbation moment by rapidly increasing CW rotation until reaching maximum within the same left stance phase immediately after perturbation. Likewise, when perturbation was applied in CCW direction CCW rotation increased substantially. On the other hand, neither of the two perturbations caused any substantial pelvis displacements in frontal or sagittal planes.

Compared to perturbations in sagittal and frontal planes perturbations in transversal plane had minor effect on stepping responses (Fig. 6). After perturbation in CW direction the first step after perturbation was slightly shorter whereas in the following steps step lengths settled in the proximity of step length of unperturbed walking. In ML direction we notice increase in step width in first three steps after perturbation which indicates three consecutive more outward foot placements. On the other hand CW perturbation did not have any substantial effect on step time. The effect of CW perturbation on step length and step width is graphically illustrated with footprints in Fig. 10. Statistical analysis showed that after perturbation in CW direction only step length and step width responses changed significantly in observation period but not also step time responses and post-hoc pairwise analysis found that statistically significant interactions between steps in observation period exist in step length and step width responses. Main effects and pairwise interactions are listed in Table 2.

When perturbation was applied in CCW direction changes in step length, step width and step time responses are comparable to those of unperturbed walking. The effect of CCW perturbation on step length and step width is graphically illustrated with footprints in Fig. 11. Statistical analysis found no statistically significant changes in step length, step width or step time responses in observation period only after perturbation in CCW direction and in unperturbed walking. Main effects are listed in Table 2.

## Discussion

### Design and control characteristics

In relation to existing devices for gait rehabilitation design of BAR is specific in that BAR promotes balance training during overground walking, whereas other approaches integrated treadmill into their designs. There is an ongoing debate in the literature as to whether treadmill walking and overground walking are similar. Some studies have shown that treadmill may elicit somewhat different gait kinematics as opposed to overground walking [25, 26]. Other studies argue that overground walking and treadmill walking produce equal kinematic and kinetic data as long as treadmill speed is constant and subjects are familiarized with the treadmill [27, 28]. One distinctive research interest that could be thoroughly investigated with BAR but not also with treadmill based balance assessment devices are balance responses associated with changing direction of walking. According to some studies changing direction represents up to half of all walking activities [29] and demands different kinematic as well as spatio-temporal gait characteristics [30] than straight walking. Furthermore, compared to straight walking people increase or decrease recruitment of certain muscle groups when changing direction [31, 32] which requires specific control strategies. Balance mechanisms associated with changing direction and turning are poorly understood and are yet to be investigated.

One key design component that should be of great concern when assessing balance during walking is unconstrained pelvis movement. In the present study we have shown that integral part of balance responses is the interplay between stepping responses, weight transfer, forward propulsion, CoM dynamics as well as time component that is exercised in pelvis in all planes of movement. Additionally [33] has shown that fixating the pelvis by reducing its DoF changes gait characteristics and may even impede proper execution of balance responses. In this sense innovative design of BAR which promotes six DoF in pelvis enables the user to properly utilize mechanisms associated with dynamic balance. Out of six DoF three DoF are actuated and admittance controlled so that BAR in those three DoF manifests itself to the user as a transparent device, meaning that the interaction forces/moment were small and did not significantly interfere with subjects’ movement. In the remaining three DoF subjects were subjected only to the spring-balanced weight and the inertial properties of PE which had minor effect due to lightweight construction. To what extent degrees of freedom of PM liberate pelvis movement was verified in unperturbed walking. When qualitatively comparing alternating ML and AP pelvis movement and normative data from the literature [1] we notice that both share similar sinusoidal pattern. Similarly, in perturbed walking six DoF in pelvis combined with transparent operation of admittance controller offered the subject to freely react to perturbation with necessary pelvis displacement or rotation.

Furthermore, avoiding position control as a key control strategy in rehabilitation robots and substituting it with admittance control offers several rehabilitation implications, one being “assist as needed” paradigm. Contrary to position control where the measured kinematics is fed into predefined gait pattern, “assist as needed” paradigm personalizes cooperation between the subject and the robot in a sense that the robot in order to enhance voluntary participation of subject first evaluates subject’s performance and then supplies as much support as particular subject needs to accomplish selected task. Characteristics of “assist as needed” robot operation greatly depend on the characteristics of associated force control. In our study, when subjects were walking in “transparent mode” and were not subjected to external perturbation forces (BAR was following subject’s pelvis) low standard deviation in pelvis movement and interactions forces/moment indicate constant and repeatable interaction conditions. Furthermore when subjected to perturbation forces variability in responses remained similar to variability of unperturbed walking indicating high repeatability of experimental conditions. This implies that that BAR is capable of supplying desired force fields in selected actuated DoF that are very repeatable in timing as well as in amplitude and therefore has strong potential for successful implementation of “assist as needed” robot operation.

### Responses to selected perturbations

When subjects were subjected to perturbations they had to confront with unexpected errors in CoM acceleration and velocity and associated disharmony between CoM and center of pressure (CoP). These error patterns differed greatly depending on perturbation direction and as such they were handled with strategies that depended on perturbation direction.

#### Perturbations in frontal plane

Perturbations in frontal plane at the time of left foot contact caused the CoM to accelerate into direction of perturbation force, i.e. left and toward the stance leg after perturbation to the left vs. right and away from the stance leg after perturbation to the right. In general depending on the amplitude of perturbation force such situations people handle by employing “lateral ankle strategy” [34] if pushes are mild or combine “lateral ankle strategy” with dominating “stepping strategy” [18] when vigorous perturbations demand intervention that exceed the capacity of “lateral ankle strategy”, that is when “lateral ankle strategy” due to a small foot width of supporting leg cannot produce enough lateral moment that would prevent the projection of CoM from progressing and accelerating beyond or away from CoP [19]. Stepping responses and foot print illustrations (Figs. 5 and 7) therefore suggest that when perturbed in frontal plane subjects were forced to employ “stepping strategy” to regain stability. Different implications were observed depending whether perturbation was employed to the left or to the right. Perturbation to the left elicited substantially decreased step width which indicates placement of right foot in front of the left stance foot (in some cases right leg crossed over left stance leg, resulting in negative step width) and adjustment in the next left foot contact which was placed even more to the left to acknowledge new CoM position. Similar mechanism was observed by [18] where placing the swing foot in front of the stance foot was referred to as the “inward strategy”. On the other hand reaction to perturbations to the right involved more outward right foot placement which is in agreement with existing studies where such mechanism is referred to as an “outward strategy” [18]. In both cases the following large step in the opposite direction are to be associated to subjects proactive attempts to restore original line of walking. In addition, we also observed that perturbations in frontal plane induced considerable and statistically significant adjustments associated with sagittal plane spatial and temporal parameters i.e. step length and step time respectively. Regardless whether subjects were subjected to perturbation to the left or to the right first step was in both cases substantially shorter and faster, especially short was the step time after perturbation to the right. Similar mechanism was observed in [18] where shorter stride time was found for left pushes at right foot contact that was attributed to shorter swing time of the contralateral leg. Afterwards, while step time in both cases normalized, step length remained shorter when subjects were perturbed to the left but was normalized when they were perturbed to the right. Therefore it seems that subjects required two steps to respond to perturbation to the left as opposed to only single step response when they were perturbed to their right. One explanation for such strategy might be that people tend to apply “stepping strategy” first to establish favourable relation between projection of CoM, CoM velocity and CoP that would then allow application of “lateral ankle strategy” for fine tuning lateral balance. Therefore, compared to existing studies that focused only on mechanisms associated with frontal plane [18, 19], our results suggest that stepping strategy associated with perturbations in frontal plane is not limited only to the frontal plane but should be decomposed into both frontal contributions as well as sagittal and temporal contributions.

#### Perturbations in sagittal plane

Similar to perturbations in frontal plane perturbations in sagittal plane accelerated CoM in the direction of perturbation force. Footprint illustrations and stepping responses (Figs. 8 and 9) show that perturbations in sagittal plane did not evoke substantial adjustments in frontal plane, more noticeable effect was noted in step length responses but the major effect was observed in step time of the first step immediately after perturbation. We may argue that when perturbed in sagittal plane “ankle strategy” in AP direction (hence “posterior ankle strategy”) has considerably more potential to affect the relation between projection of CoM, CoM velocity and CoP, than “lateral ankle strategy” after perturbation in frontal plane. On one hand this could be attributed to relatively longer foot length compared to small foot width. On the other hand this is also related to fundamentally different function of sagittal movement compared to frontal movement. Namely it is in domain of sagittal plane to handle forward progression by controlling forward propulsion and braking which is to large extent controlled by ankle plantar flexor activation during push off and during loading response. To increase forward momentum high activation of plantar flexor muscle group generates more plantar flexor moment and vice versa, to decrease forward momentum their low activation generates less plantar flexor moment during push off and more plantar flexor moment during early stance. These mechanisms have immediate impact on the dynamics of CoP, i.e. large plantar flexor moment causes anterior CoP position whereas low plantar flexor moment causes more posterior CoP position. We assume that when perturbed in sagittal plane people fully exploit this mechanisms. To compensate for increase in forward momentum of CoM after perturbation in forward direction one may first increase braking on stance leg followed by plantar plexor activation which reduces forward propulsion associated with push off. Likewise to compensate for decrease in forward momentum of CoM after perturbation in backward direction one may first decrease braking during early stance, prolong stance time and later increase activation of ankle plantar flexor to increase forward propulsion associated with push off. We found no other studies that have investigated balance mechanisms associated with pelvis perturbations in sagittal plane that we could relate our conclusions to. Therefore to substantiate our findings and assumptions further studies are required that would also measure ground reaction forces.

#### Perturbations in transversal plane

Contrary to changes in linear momentum that is produced during linear perturbations in frontal and sagittal planes, angular perturbations in transversal plane enforce changes in angular momentum. Consequently angular perturbations in transversal plane do not accelerate CoM in any direction but cause pelvis rotation in the direction of perturbation. For this reason we observe profound pelvis movement in transversal plane but very small changes in movement in sagittal and frontal planes that is comparable to unperturbed movement in sagittal and frontal movement respectively. Also, except from statistically significant shorter steps and longer step times following CW perturbation - that are small in amplitudes - footprint illustrations and stepping responses show that angular perturbations do not interfere with balance substantially in sagittal and frontal planes.

### Limitation of study

Very common limitation associated with studies that involve overground walking is the inability to measure also ground reaction forces which would further elaborate and substantiate mechanisms associated with balance. Usually necessary equipment is confined to relatively small gait labs with embedded force plates in the center of walkway where ground reaction forces are measured once per trial and only in limited number of trials. Taking into account the number of perturbations we employed as well as the number of steps we considered to be associated with each response repeating such experiment in gait lab would be impossible. For this reason similar studies are often conducted on instrumented treadmill with embedded force sensors that allow ground reaction force and CoP recordings. In our future studies we intend to place BAR onto instrumented treadmill in order to compare balancing responses during overground and treadmill conditions.

Limitation of presented study is associated also to investigating only a single onset of perturbation with respect to phase of walking. In our study subjects were presented with perturbations immediately after foot strike of the left leg whereas we could assume that same perturbations applied in different phases of gait would result in different balance responses. Namely extensive studies of the biomechanics of human walking have shown that gait is composed of several fundamental mechanisms (e.g. weight acceptance, weight transfer, forward propulsion...) that are executed in specific phases of gait cycle. Taking into account the specific function of each mechanism in gait we may assume that interfering with any of them would evoke specific balance responses. Further studies are planed to substantiate these assumptions.

## Conclusions

In this paper we describe an innovative design of a novel balance assessment robot (BAR). To the best of our knowledge BAR is the only device that can explore balance responses following proximal perturbations during overground walking. In this research we explored mechanical and control characteristics of BAR and used BAR to evoke and identify normative balance responses in neurologically healthy individuals by applying proximal perturbation forces/moments in transversal plane of movement. Results indicate that the proposed device can repeatedly reproduce the same experimental conditions. Results also suggest that “stepping strategy” is the dominant strategy for coping with perturbations in frontal plane, perturbations in sagittal plane are to greater extent handled with “ankle strategy” while angular perturbations in transversal plane do not pose substantial challenge for balance. Results also show that specific perturbations in general elicit responses that are not necessarily confined to the plane of perturbation but could extend also to other planes of movement that are not directly associated with plane of perturbation as well as to spatio temporal parameters of gait.

Encouraging results of present study provide strong motivation for future investigation of postural responses also in neurologically impaired subjects. We also speculate that BAR could be in future used in clinical environment as an effective training method for improving balance during walking. The design characteristics of BAR that ensure unconstrained pelvis movement that can be adjusted trough different operating modes of PM to achieve desired and subject specific supporting regimes either during walking or following perturbations could be widely beneficial in clinical practice. We envisage that such system could be used in initial phases of gait rehabilitation as a fall safe training device for recovering elementary gait mechanisms associated with forward propulsion, weight transfer, cyclical leg movement and dynamic balance during straight walking or while practising complex walking manoeuvres (e.g. turning, starting, stopping,...) where haptic support would be adjusted according subjects needs in an “assist as needed” manner. In later phases of gait rehabilitation subjects could be subjected to perturbation training where BAR would be used as a mean to deliver perturbations as well as to provide assistive forcefield that would in an “assist as needed” manner support subjects in their effort to regain balance after perturbations, thus learning proper balance mechanisms. We hypothesize that once balance mechanisms improved subjects would inherently respond more efficiently also in fall threatening situations thus reducing chances of falling.

## References

[1] Winter DA (1998). The Biomechanics and Motor Control of Human Gait: Normal, elderly and Pathological.

[2] Blicher JU, Nielsen JF (2009). Cortical and spinal excitability changes after robotic gait training in healthy participants. Neurorehab Neural Re.

[3] Classen J, Liepert J, Wise SP, Hallet M (1998). Rapid plasticity of human cortical movement representation induced by practice. J Neurophysiol.

[4] Schabruna SM, Riddingb MC, Chipchasea LS (2013). An update on brain plasticity for physical therapists. Physiother Pract Res.

[5] Woollacott MH, Tang PF (1997). Balance control during walking in the older adult: research and its implications. Phys Ther.

[6] Tang PF, Woolacott MH, Chong RK (1998). Control of reactive balance adjustments in perturbed human walking: roles of proximal and distal muscle activity. Exp Brain Res.

[7] Marigold DS, Bethune AJ, Patla AE (2005). The role of the swing limb and arms in the reactive recovery response to an unexpected slip during locomotion. J Neurophysiol.

[8] Schillings AM, Wezel BMH, van Mulder T, Duysens J (2000). Muscular responses and movement strategies during stumbling over obstacles. J Neurophysiol.

[9] Pijnappels M, Bobbert MF, Van Dieen JH (2006). Emg modulation in anticipation of a possible trip during walking in young and older adults. J Electromyogr Kinesiol.

[10] Schillings AM, Mulder T, Duysens J (2005). Stumbling over obstacles in older adults compared to young adults. J Neurophysiol.

[11] Ferber R, Osternig LR, Woollacott MH, Wasielewski NJ, Lee JH (2002). Reactive balance adjustments to unexpected perturbations during human walking. Gait Posture.

[12] Bhatt T, Wang TY, Yang F, Pai YC (2013). Adaptation and generalization to opposing perturbations in walking. Neuroscience.

[13] Pai YC, Bhatt T, Wang E, Espy D, Pavol MJ (2010). Inoculation against falls: rapid adaptation by young and older adults to slips during daily activities. Arch Phys Med Rehabil.

[14] Bieryla KA, Madigan ML (2011). Proof of concept for perturbation-based balance training in older adults at a high risk for falls. Arch Phys Med Rehabil.

[15] Shapiro A, Melzer I (2010). Balance perturbation system to improve balance compensatory responses during walking in old persons. J Neuroeng Rehabil.

[16] Wang TY, Bhatt T, Yang F, Pai YC (2012). Adaptive control reduces trip-induced forward gait instability among young adults. J Biomech.

[17] Shinya M, Fujii S, Oda S (2009). Corrective postural responses evoked by completely unexpected loss of ground support during human walking. Gait Posture.

[18] Hof AL, Vermerris SM, Gjaltema WA (2010). Balance responses to lateral perturbations in human treadmill walking. J Exp Biol.

[19] Hof AL, Duysens J (2013). Responses of human hip abductor muscles to lateral balance perturbations during walking. Exp Brain Res.

[20] Ichinose WE, Reinkensmeyer DJ, Aoyagi D, Lin JT, Ngai K, Edgerton RV, Harkema SJ, Bobrow JE (2003). A robotic device for measuring and controlling pelvic motion during locomotor rehabilitation. Proceedings of the 25th Annual International Conference of the IEEE EMBS: 17-21 September 2003.

[21] Pietrusinski M, Cajigas I, Mizikacioglu Y, Goldsmith M, Bonato P, Mavroidis C (2010). Gait rehabilitation therapy using robot generated force fields applied at the pelvis. Proceedings of IEEE Haptics Symposium: 25-26 March 2010.

[22] Vashista V, Jin X, Agrawal SK (2014). Active tethered pelvic assist device (a-tpad) to study force adaptation in human walking. Proceedings of IEEE International Conference on Robotics and Automation (ICRA): May 31 - June 7 2014.

[23] Perry J, Garrett M, Gronley JK, Mulroy SJ (1995). Classification of walking handicap in the stroke population. Stroke.

[24] Hak L, Houdijk H, Steenbrink F, Mert A, van der Wurff P, Beek PJ, van DieËn JH (2012). Speeding up or slowing down?: Gait adaptations to preserve gait stability in response to balance perturbations. Gait Posture.

[25] Aaslund MK, Moe-Nilssen R (2008). Treadmill walking with body weight support effect of treadmill, harness and body weight support systems. Gait Posture.

[26] Alton F, Baldey L, Caplan S, Morrissey MC (1998). A kinematic comparison of overground and treadmill walking. Clin Biomech.

[27] Riley P, Paolini G, Della Croce U, Paylo KW, Kerrigan DC (2007). A kinematic and kinetic comparison of overground and treadmill walking in healthy subjects. Gait Posture.

[28] Lee JS, Hidler J (2008). Biomechanics of overground vs. treadmill walking in healthy individuals. J Appl Physiol.

[29] Glaister BC, Bernantz GC, Klute GK, Orendurff MS (2007). Video task analysis of turning during activities of daily living. Gait Posture.

[30] Orendurff MS, Segal AD, Berge JS, Flick KC, Spanier D, Klute GK (2006). The kinematics and kinetics of turning: limb asymmetries associated with walking a circular path. Gait Posture.

[31] Ventura JD, Klute GK, Neptune RR (2015). Individual muscle contributions to circular turning mechanics. J Biomech.

[32] Courtine G, Schieppati M (2003). Human walking along a curved path. II. Gait features and EMG patterns. Eur J Neurosci.

[33] Veneman JF, Menger J, van Asseldonk EH, van der Helm FC, van der Kooij H (2008). Fixating the pelvis in the horizontal plane affects gait characteristics. Gait Posture.

[34] King DL, Zatsiorsky VM (2002). Periods of extreme ankle displacement during one-legged standing. Gait Posture.

